# Book Reviews

**Published:** 1896-12

**Authors:** 


### Book Reviews.

A TREATISE ON SURGERY. By American authors. Edited by ROSWELL PARK, M. D., Professor of Surgery and Clinical Surgery at the Medical Department of the University of Buffalo. In two octavo volumes, comprising 1,603 pages, with 807 engravings and 38 full-page plates in colors and monochrome. Volume I., General Surgery and Surgical Pathology. Volume II., Special Surgery. Philadelphia: Lea Brothers & Co. 1896.

The literature of surgery is especially rich in quantity and quality at the present time. Many new treatises have been added to it within the last few years, while many old ones have undergone revisement in new editions put forth. Whenever, therefore, a new candidate for professional favor appears it is pertinent to scrutinise its claims to literary and scientific preferment. In the present instance we shall examine the work all the more impartially because the editor, who is also in great part author, is a resident of our own city where he has achieved fame as a teacher and writer.

At the outset let us call attention to the fact that a number of departments have recently been added to the surgical field, so that a treatise of today differs from that of yesterday in many essential particulars. This fact has been conspicuously held up to view by Park in the work now under consideration. The first part of Volume I. is devoted to the consideration of surgical pathology, the five chapters of which are written by the editor himself. This is one of the departments in which this work may properly claim advantage over many others. Hyperemia being the first step in pathological change, receives appropriate discourse in the first chapter of the book. It is a condition too little understood and too often confounded with congestion in type or speech, in ignorance or carelessness. The minute pathology of the blood is a field that has lately demanded increasing study and research. The importance of a knowledge of blood pathology is of constantly enlarging importance to the surgeon, and he will gain light on the subject from this chapter. Inflammation is always an engaging field of surgical research and an intricate knowledge of its subtle and various manifestations is essential to surgical skill. The chapter on ulceration and gangrene presents the interesting and important features of these subjects.

The second part, devoted to surgical diseases, opens with a chapter on autoinfection. Here is one of the most absorbing subjects presented in modern surgical pathology. Park emphasises the importance of a thorough understanding of autointoxication, asseverating that among the most predisposing causes of what may a little later appear as distinct surgical sepsis are retained excrementitious products. That enterosepsis may be mistaken for surgical fever cannot be too strongly accentuated, and is a point which the author makes clear. The lesson this chapter teaches is the practical one of careful preparation of patients for surgical procedures, a subject so often loosely insisted upon in magazine papers and discussions, but for which here are given substantial and convincing reasons. The surgical fevers and septic infections naturally come next, and these are followed by two chapters on surgical diseases common to man and the domestic animals. These all possess intense interest and are discussed in the light of the newest pathology. The editor is the author of these four chapters, together with an appendix to Chapter IX. on leprosy.

The next following chapters on syphilis and gonorrhea are profitable and deserve special remark with reference to their illustrations, particularly plates VIII. and IX.—early rupial syphilide and tubercular ulcerating syphilide respectively—the latter showing lesions in different stages.

Shock and collapse always present questions of the deepest concern to the surgeon. The editor contributes but a brief chapter to their consideration. The question of immediate operation after shock often calls for the keenest surgical judgment. We feel disposed to challenge the use of the word "diagnosed," as it occurs in this chapter and other places in the work. "Diagnosticated" is more euphonious, if not better English. The next four chapters,

XIII., XIV., XV. and XVI.—Scurvy and rickets, Surgical aspects and sequelæ of other infections and diseases, Poisoning by animals and plants, and Acute intoxications, including delirium tremens—are also contributed by the editor. It seems rather odd to find delirium tremens considered in a surgical text-book. Nevertheless, it is a disease that often complicates surgical cases, and this the author considers a sufficient reason for dealing with it in this place, an opinion in which we fully concur.

Coming now to Part III., we find surgical principles and methods, together with minor procedures, adequately set forth. The first two chapters, one dealing with hemorrhage and the other with minor surgery and bandaging, are written by our fellow-townsman, Prof. Parmenter. Without entering into detail, we may observe that we have rarely seen as much useful information condensed into as little space, all of which will prove of special advantage to the young surgeon. The chapter on anesthesia and anesthetics singularly enough is not contributed by a surgeon, but no one will question the ability of Hobart Amory Hare to deal with the subject in an able manner. Perhaps, too, it were better to turn on the light from the medical and therapeutical standpoints, since these departments must be invoked in case of threatened anesthetic danger.

Surgical diagnosis calls for the profoundest surgical knowledge and skill, as well as the ripest experience. A chapter on this subject is well presented by Dr. Chauncey P. Smith, also of Buffalo.

Under the head of Injury and repair, to which Part IV. is devoted, we find wounds, gunshot wounds, processes of repair, treatment of wounds (antisepsis and asepsis), considered by Prof. Charles B. Nancrede, in four chapters. They are all well written and are characterised by experience in teaching and a thorough knowledge of the subjects.

Surgical affections of the tissues and tissue-systems forms the title of Part V., and the opening chapter on cysts and tumors is contributed by the editor. Park's classification of tumors is the best we have seen. If we had space at command we should like to treat of this part of the work in some detail, since its importance would seem to merit extended comment. We must, however, content ourselves with simply calling attention to this section of the text-book in which Park shows originality of classification and skill in dealing with the subject. The tables of differentiation between benign and malignant growths on pages 440-441 are unusually complete, and will be found valuable aids to diagnosis. It cannot be too often accentuated that early diagnosis is of the greatest importance in suspected malignancy, and that a grave responsibility in this regard rests upon the general practitioner of medicine as he usually first sees these cases.

Surgical diseases of the skin afford Prof. Hardaway an opportunity to present some interesting text, which is well illustrated. Another short chapter by Prof. Parmenter covers the essential ground in relation to burns, scalds and frost-bites. A very entertaining section is contributed by Dr. Burrell on affections of the muscles, tendons, tendon-sheaths, bursæ and fasciæ. This is a most practical chapter and one that deserves to be studied by every young physician. It is illustrated with rare completeness. Injuries and diseases of the lymphatic vessels and nodes by Prof. Gerrish; Surgical injuries and diseases of the veins by Prof. Holloway; Surgical injuries and diseases of the arteries by Prof. Eve; Injuries of the joints and joint structures and operations on joints by Prof. Ransohoff; Surgical diseases of the osseous system by Prof. Park; Fractures and dislocations by Prof. Mudd, constitute the remaining chapters in this volume.

The second volume is devoted to the consideration of special or regional surgery. The opening chapter deals with surgical diseases and injuries of the head, is written by the editor, is a field in which Prof. Park has heretofore displayed great knowledge, and in it will be found recorded his latest and ripest experience. The illustrations, speaking generally, are new and unique.

The next chapter considers injuries and surgical diseases of the spine, and is written by Edward H. Bradford, whose name is well known in orthopedic literature. Here, too, both text and illustrations are of absorbing interest. Surgical diseases and injuries of the heart and pericardium, with surgery of the large blood-vessels, including ligations, forms the subject of a chapter written by Prof. Eve. This and the following chapter on surgical diseases and injuries of the respiratory organs, by Prof. Delavan, contain interesting data, cases and illustrations. Two chapters on surgical diseases and injuries of the face and neck are contributed by Prof. Souchon, in which are found some novelties, including Dr. G. W. Wende's case of rhinoscleroma, first published in the JOURNAL, March, 1896, p. 642.

We now come to a chapter on surgery of the chest, written by Prof. Dennis, which embraces the present status of this branch of new surgery. It is one of the best presentations of the subject we have seen. Following it are considered surgical diseases and injuries of the mouth, tongue, teeth and jaws, by Prof. Bevan, to which is added an appendix by Dr. Brophy on early operations for closure of cleft palate. Surgery of the abdomen, which now is of such absorbing interest, is jointly written by Drs. Richardson and Cobb. The former also contributes the chapter on hernia, which is in every way an admirable presentation of the subject. Diseases of the rectum and sigmoid flexure, by Kelsey, and genito-urinary surgery, by Belfield, follow in their natural order of sequence, and then comes a short chapter on chancroid by the editor.

The next chapter is devoted to the consideration of surgical diseases and injuries of the female reproductive organs, and is written by Prof. Etheridge. It would seem at first glance that rather a limited space had been given in this instance to the consideration of a very large subject. Again, some might think that in view of such limitation the space might have been used to better advantage. On the whole, however, our conclusion is that in a strictly surgical work the author of this chapter has improved his opportunity to good advantage. Surgical diseases and injuries of the breast are next dealt with by Prof. Parker, and then follows amputations by Prof. Matas. Orthopedic surgery proper is considered in a chapter by Dr. Lovett. This is a branch of surgery in which there is constantly increasing interest, and this author deals with it in a scientific manner. He has illustrated his chapter with an admirable discretion that serves to strengthen his instructive text. In the next chapter we find plastic surgery treated of by Dr. Gerster, who illustrates his paper with numerous schematic drawings. Next following is a chapter on surgical injuries and diseases of the eye and orbit, by Charles Stedman Bull; then comes one on surgical injuries and diseases of the ear, by Prof. Blake. The final chapter is written by the editor himself, and is devoted to the consideration of skiagraphy, or the application of the Röntgen rays to surgery. It is a résumé of whatever is known of a subject still under investigation, and is illustrated with some beautiful plates.

The claim of this work to establish itself as a text-book justifies the assertion that, though it will not be likely to supplant some of the individual treatises that have become classics, it nevertheless presents so much that pertains to new surgery, and withal offers it to the profession in such a new way, as to deserve to be placed by their side, supplementing and complementing them in a useful manner. The rhetoric of the work merits remark for its excellence and the proof-reading has been of a quality to challenge approval, the errors being very few and insignificant. A reference made by Prof. Eve, Vol. II., page 127, should be corrected to read "BUFFALO MEDICAL JOURNAL, January, 1852, Vol. VII., page 479."

In concluding this imperfect résumé, the object of which has been to comment on the special features of the work rather than to critically review the whole, we may summarise those that stand out conspicuously as follows:

I. The pathology which it teaches, and especially that set forth regarding autointoxications, together with its classification of tumors.

II. Originality, both of cases cited and of illustrations, as well as the large number of each.

III. The stress laid upon diagnosis and the excellent treatment of this important department throughout the work.

IV. The fact that each volume can be purchased separately should be noted, though we doubt if it is a privilege that will often be exercised.

V. And, finally, the general arrangement and dress of the volumes—a part in which the publishers deserve to share praise.

The treatise, as a whole, is a credit to American surgery, and especially to the editor and his excellent staff of collaborators.

ANATOMY, DESCRIPTIVE AND SURGICAL. By HENRY GRAY, F. R. S., Lecturer on Anatomy at St. George's Hospital, London. New and thoroughly revised American edition, much enlarged in text, and in engravings, both colored and black. In one imperial octavo volume of 1239 pages, with 772 engravings on wood. Price of edition with illustrations in colors, cloth, $7; leather, $8. Price of edition with illustrations in black, cloth, $6; leather, $7. Philadelphia and New York: Lea Brothers & Co., Publishers. 1896.

It is always an agreeable duty to write a notice of Gray's Anatomy. It has become a general favorite with teachers and students, and it will long remain so, we doubt not, while it continues on its present lines of clear and intelligent presentation of a subject that must form the foundation to an acquirement of medical knowledge.

This edition has been revised exclusively by American anatomists, whose aim has been to thoroughly adapt it to the requirements of teachers and students of the present day. Certain departments have undergone complete change, necessitated by the advances that have recently taken place in them. This is especially the case with those on the brain, the teeth and the abdominal viscera, histology and development.

The illustrations in Gray's Anatomy have always been one of its especial features ; each bone, ligament, muscle, nerve, artery and tissue has been appropriately labeled, and in late editions have appeared in colors where essential. In this edition 135 engravings have been added, bringing the aggregate up to a total greater than in any other anatomical work. Moreover, their quality is not second to any, a matter of paramount importance in such a treatise.

We have no hesitation in saying that, taken all in all, Gray's Anatomy affords the student more satisfaction than any other similar treatise with which we are familiar.

---

I. THE MEDICAL NEWS VISITING LIST FOR 1897. Weekly (dated, for 30 patients) ; Monthly (undated, for 120 patients per month) ; Perpetual (undated, for 30 patients weekly per year) ; and Perpetual (undated, for 60 patients weekly per year). The first three styles contain 32 pages of data and 160 pages of blanks. The 60-patient Perpetual consists of 256 pages of blanks. Each style in one wallet-shaped book, with pocket, pencil and rubber. Seal Grain Leather, $1.25. Philadelphia and New York : Lea Brothers & Co.

II. THE MEDICAL RECORD VISITING LIST AND PHYSICIANS' DIARY FOR 1897. New revised edition. With Calendar, Tables of Doses, Tables of Equivalents, Directions for Emergencies, Antisepsis, Disinfection, Special Memoranda, Cash Account, etc., etc., thirty and sixty patients per week, bound in black or red morocco leather, with flap, $1.25 and $1.50. Circular on application. New York : William Wood & Company, Publishers.

III. THE PHYSICIANS' VISITING LIST (Lindsay & Blakiston) FOR 1897. Forty-sixth year of its publication. Sold by all booksellers and druggists. Philadelphia : P. Blakiston, Son & Co., 1012 Walnut street.

I. The *Medical News* visiting list is the first to make its appearance for the new year. It has been thoroughly revised for 1897, and is in every way adapted to the requirements of the present period. Thirty-two pages of the book are set apart for such useful data as the busy physician may stand in need of, such as an alphabetical table of diseases, a list of approved remedies, a table of doses, together with sections on examinations of urine, artificial respiration, incompatibles, poisons and antidotes, diagnostic table of eruptive fevers and the ligation of arteries. The mechanical execution of the book, both as to material and quality, is unexceled and it is supplied with a ready reference thumb-letter index when desired at an additional cost of 25 cents. This standard visiting list is one of the best in the market.

II. The second in order of arrival is the well-known *Medical Record* visiting list. This, too, has been revised to meet the requirements of the year 1897. The thirty closely-printed pages that precede the visiting list proper constitute a ready reminder on many subjects that are needful in every-day practice, but that need to be referred to frequently in order to be kept fresh in thought. The book before us is arranged for sixty patients a week, yet is so compact in form that it is not cumbrous in the pocket. Those who have been in the habit of using this visiting list will easily find themselves in favor with the new edition; and no doubt many new purchasers will be found to swell the aggregate sales beyond any previous figures.

III. The old-time favorite visiting list of Lindsay & Blakiston, now in the forty-sixth year of its publication, presents itself in its usually complete, compact and useful form. For the year 1897 it is issued as heretofore in three editions. The first or regular edition is arranged for from twenty-five to one hundred patients a week. For fifty patients and above it is issued in two volumes. A perpetual edition is printed like the regular, but without dates, so that it can be commenced at any time and used until full. A monthly edition, designed for the convenience of those who wish a new book every month. This visiting list is unsurpassed in practical usefulness.

---

An American Text-Book of Applied Therapeutics for the Use of Practitioners and Students. Edited by J. C. Wilson, M. D., Professor of the Practice of Medicine and Clinical Medicine in the Jefferson Medical College; Attending Physician to the Hospital of the Jefferson Medical College, to the German Hospital and to the Pennsylvania Hospital, Philadelphia. Assisted by Augustus A. Eshner, M. D., Professor of Clinical Medicine in the Philadelphia Polyclinic; Attending Physician to the Philadelphia Hospital. Octavo, pp. 1326. Illustrated. Price, cloth, $7; sheepskin and half morocco, $8; half Russia, $9.50. Philadelphia: W. A. Saunders, 925 Walnut street. 1896.

The plan of this work is unlike that of the ordinary treatise on therapeutics. It is a grouping of a series of separate articles on each subject chosen and, generally speaking, each author has confined himself to a single monograph. The exceptions are, Atkinson, John C. Da Costa, Augustus A. Eshner, James Stewart, George Dock, Edwin E. Graham, Wm. C. Dabney, J. T. Eskridge, Wharton Sinclair, Guy Hinsdale, Frederick A. Packard and the editor, Dr. J. C. Wilson. The article on Diseases of the stomach: duodenal ulcer, is written by Dr. Charles G. Stockton, of Buffalo. It is a concise setting forth of the therapy of this important group of diseases, and will be referred to by physicians who desire to keep pace with the literature on this subject.

It would be a difficult matter to present a detailed review of this book. To carefully analyse each one of its seventy-eight sections would consume more time and occupy more space than can now be devoted to the work. However, it is only just to say that it is one of the most complete books of reference that has been presented to the profession on medicine in a long period of time; and never before have we had one that undertook to cover the field in this manner. The editor is an experienced teacher, as well as a man of literary accomplishments. He deserves the thanks of physicians for bringing out this treatise.

---

THE AMERICAN ACADEMY OF RAILWAY SURGEONS. Report of the second annual meeting, held at Chicago, Ill., September 25, 26 and 27, 1895. Edited by R. HARVEY REED, M. D., Columbus, O. Chicago: American Medical Association Press. 1896.

This organisation has established itself on a substantial basis and presents its work in an attractive though modest volume. It is classified into chapters and is properly indexed so that its contents become easily accessible to whoever may search for its interesting material. A number of illustrations appear in the book, including which are half-tone portraits of the officers of the academy. Among the latter we observe an excellent reproduction of a photograph of Dr. C. M. Daniels, of Buffalo, who was first vice-president last year and is a member of the executive board at present. One of the good features of this volume is the appearance of the discussions in excellent form.

---

EIGHTEENTH ANNUAL REPORT OF THE STATE BOARD OF HEALTH OF THE STATE OF ILLINOIS. (Being for the year ended December 31, 1895.) With an Appendix containing the Official Register of Physicians and Midwives. 1896. Springfield, Ill.: Ed. F. Hartman, State Printer. 1896.

The Illinois State Board of Health under the directorship of the sturdy and accomplished Rauch enjoyed the confidence and respect of the entire medical profession, but we regret to say that of late its influence has deteriorated. This we attribute to political conditions that naturally arise in a state which has for its governor such a man as John P. Altgeld. We make no reflections on the personnel of the board as at present constituted.

The volume before us contains the official register of physicians and midwives for 1896, in which its chief interest centers.

